# Application of I-Optimal Design for Modeling and Optimizing the Operational Parameters of Ibuprofen Granules in Continuous Twin-Screw Wet Granulation

**DOI:** 10.3390/biomedicines11072030

**Published:** 2023-07-19

**Authors:** Jie Zhao, Geng Tian, Haibin Qu

**Affiliations:** Pharmaceutical Informatics Institute, College of Pharmaceutical Sciences, Zhejiang University, Hangzhou 310058, China; zhaojie_1021@zju.edu.cn (J.Z.); iamtiangeng@zju.edu.cn (G.T.)

**Keywords:** twin-screw wet granulation (TSWG), I-optimal design, continuous manufacturing, process understanding, design space

## Abstract

The continuous twin-screw wet granulation (TSWG) process was investigated and optimized with prediction-oriented I-optimal designs. The I-optimal designs can not only obtain a precise estimation of the parameters that describe the effect of five input process parameters, including the screw speed, liquid-to-solid (L/S) ratio, TSWG feed rate, and numbers of the 30° and 60° mixing elements, on the granule quality in a TSWG process, but it can also provide a prediction of the response to determine the optimum operating conditions. Based on the constraints of the desired granule properties, a design space for the TSWG was determined, and the ranges of the operating parameters were defined. An acceptable degree of prediction was confirmed through validation experiments, demonstrating the reliability and effectiveness of using the I-optimal design method to study the TSWG process. The I-optimal design method can accelerate the screening and optimization of the TSWG process.

## 1. Introduction

The pharmaceutical industry is currently undergoing a paradigm shift from traditional batch production to continuous manufacturing (CM) [1,2]. Twin-screw wet granulation (TSWG) is a typical method of continuous granulation; it has the advantages of a high processing volume, higher production efficiency, short residence time, and better mixing and controlling processes compared with traditional batch manufacturing [3]. In addition, TSWG can be readily integrated into the CM of pharmaceutical dosage forms and provides easier process scale-up, better quality assurance, low production costs, and material waste [4].

During the TSWG process, the powder and liquid binders are added at the entrance of the granulator through powder feeders and nozzles, respectively, and then the low-strength wetted agglomerates are formed and blended inside according to the conveying and mixing profile of the system. These primary agglomerates are broken apart into smaller rounded granules, allowing for growth through layering as the primary powder adheres to the surface. Uniform granules are produced by breakage and layering of the primary agglomerates under the two main rate-controlling processes [5]. In this granulation process, the critical quality attributes (CQAs) of the granules produced by TSWG are affected by many parameters. The present literature shows that the materials and binder properties, the process parameters, such as the liquid-to-solid (L/S) ratio, the rotation speed of the screw, the material feed rates, and the configuration of the screw elements (conveying elements, kneading elements, distributive elements, etc.) are the key process variables of the TSWG process [6].

An accurate model for the TSWG process would provide a thorough understanding of the process dynamics and can be used to optimize the operating conditions of the TSWG process [1]. Therefore, it is important for the process understanding to clarify the influence mechanism of the process conditions on the particle properties. Experimental studies using the design of experiment (DoE) method are typically performed to analyze the mechanism involved during the granulation process and have been extensively used to develop models to quantify the relationship between critical process parameters (CPPs) and CQAs of particles. Different DoE methods have been performed to evaluate the influence of material properties and process variables on the characteristics of granules [6,7,8,9]. Seem et al. summarized the comprehensive review of the experimental twin-screw granulator literature, indicating the complex interactions between the role of the screw element type, screw configuration, feed formulation, and liquid flow rates on the granules [10]. Liu et al. analyzed the effects of throughput, screw speed, and screw components on the properties of granules and tablets in the TSWG process using a Box–Behnken experimental design, and the design space for TSWG was defined and validated to demonstrate the robustness of the optimal operating conditions [7]. Kumar et al. used a full-factor experimental design to study the effects of process parameters (feed rate, screw speed, and L/S ratio) and equipment parameters (number of kneading elements and staggered angles) on residence time distribution, solid–liquid mixing, and final particle size distribution in the TSWG process. The results showed that it is necessary to strive for a balance between material throughput and screw speed to achieve a specific granulation time and solid–liquid mixing to achieve a high granulation yield [11]. Meng et al. studied the relationship between liquid content, throughput, and rotational speed with key performance indicators such as particle size, porosity, flowability, and particle morphology in the acetaminophen formula TSWG process using a face-centered cubic experimental design method. The results showed that the screw configuration should be fully utilized to achieve different particle characteristics [12]. It can be noticed that the previous research mainly focused on certain aspects of material properties, process parameters, or screw configurations, but it must be added that, in the case of process optimization, all aspects of the critical process parameters (CPPs) need to be considered at the same time, making it a complex process for the traditional approach that can be managed to balance the screening design and prediction optimization. In addition, the process parameters of TSWG include both discontinuous factors (e.g., screw elements configuration) and continuous factors (e.g., screw speed), and it is difficult for the common DoE method to handle.

Compared to other DoE methods, the optimal design approach can handle different types of models and experimental factors, such as continuous factors, categorical factors, and mixture factors [13,14]. It can help to obtain an improved process understanding and characterization and make predictions by the models at the same time, which provides a new and effective tool for the study of TSWG. Willecke et al. used D-optimal designs as a non-standard experimental design to investigate the impact of fillers properties (three principal components derived from eight selected pharmaceutical fillers), binder type, and binder concentration in granules together on the properties of the granule and tablet. The results showed that the filler properties mainly affected the granule characteristics, such as particle size, friability, and specific surface area, and the binder type and concentration had a relevant influence on granule flowability, friability, and compactibility [15]. Stauffer et al. used D-optimal designs to investigate the impact of raw material variability upon the granule size distribution, density, and flowability of granules produced via TSWG. Three principal components from raw material variability together with screw speed and L/S ratio were used as factors, and then the significant factors obtained from analysis were used to determine the design space of the TSWG process to reduce the active pharmaceutical ingredient (API) batch-to-batch variability [16]. Meng et al. developed an interaction model between input and output variables in the continuous TSWG of anhydrous caffeine particles using a D-optimal design and stepwise regression. Response surface design was used to study the dependence of key quality attributes of particles and tablets (D_10_, D_50_, D_90_, loose density, compacted density, and Hausner ratio) on selected key process parameters (L/S, barrel temperature, and screw speed) and screw configuration. The results showed that the impact of throughput and barrel temperature was relatively less than the L/S ratio. Higher liquid saturation leads to narrower particle size distribution, smaller porosity, enhanced flowability, and decreased tablet tensile strength, but slower drug release [17]. The usefulness of prediction-oriented optimal design criteria, such as the I-optimality criterion and the G-optimality criterion in the response surface of TSWG, is more reasonable. The I-optimality criterion can determine regions in the design space where the response falls within an acceptable range by minimizing the average variance in prediction over the design space. To build an accurate response surface in predicting the response and determining optimum operating conditions, the I-optimal design is more suitable [14]. However, the use of an I-optimal design for the process optimization of TSWG has not been reported.

This work proposed a systematic understanding of the TSWG process by the DoE method to investigate the effect of process parameters on granules properties. Ibuprofen was selected as the model drug due to its high market demand. Five process variables of screw speed, L/S ratio, powder feed rate, and screw element number (30° kneading and 60° kneading) of a twin-screw granulator were investigated using the I-optimal experimental design method. The analysis of variance was used to quantify the process response to variation in the parameters. The predictability of the developed models was validated within and without the defined design space. The I-optimal design was used for the first time to acquire better insight into the TSWG process.

## 2. Materials and Methods

### 2.1. Materials

The model formulation used in this study comprised 10% ibuprofen (Lot No. A2107096, Aladdin Biochemical Technology Co., Ltd., Shanghai, China) as API with a melting point of 75~78 °C and a density of 1.03 g/cm^3^; 40% lactose (Lot No. 1320020470) and 40% microcrystalline cellulose (MCC, Lot No. 20200719) were used as fillers (Infinitus Company Ltd., Guangzhou, China). Additionally, 10% polyvinylpyrrolidone (PVP K-30, ISP Technologies, Inc., Wayne, NJ, USA) was used as a dry binder and premixed with the raw materials using a V-mixer (Chenli powder equipment Co., Ltd., Wuxi, China). Distilled water was added as the granulation liquid.

### 2.2. Continuous Twin-Screw Wet Granulation

TSWG experiments were performed on a Pharma 11 twin-screw granulator (Thermo Fisher Scientific Inc., Waltham, MA, USA). The granulator comprised two co-rotating screws with a diameter of 11 mm and a length-to-diameter (L/D) ratio of 40:1. The Pharma 11 granulator employed the types of screw elements including conveying, kneading, and chopping elements. The pre-blend raw materials were fed into the barrel of the granulator by a single-screw feeder which was controlled by a digital governor. The relationship between the opening value (x, %) and mass flow (y, g/min) followed a linear relationship y = 0.6560x − 5.1585 (R^2^ = 0.9990). Granulation liquid was transferred to the liquid feed nozzle by a peristaltic pump (WCL Fluid Technology Co., Ltd., Changzhou, China) through silicon tubing (2.4 mm × 5.6 mm, 19 #). The relationship between the rotating speed (x, r/min) and mass flow (y, g/min) followed a linear relationship y = 0.5822x − 0.5756 (R^2^ = 0.9959).

**Figure 1:**
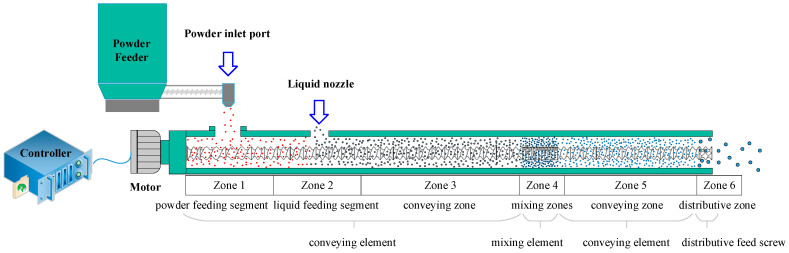
Setup and screw configuration of TSWG.

The TSWG setup is illustrated in Figure 1, which shows the Pharma 11 co-rotating parallel twin-screw granulator used for manufacturing granules. The powder inlet port was located on top of the conveying zone, and the liquid nozzle was adjacent to it.

The screw configuration is a simple system composed of conveying zone, kneading zone, and discharge zone. As the screw configuration shows in Figure 1, the conveying zone comprised two types of feed screw elements, i.e., a long-hellx feed screw (2 L/D) and a feed screw (1 L/D); the kneading zone was a combination of mixing elements in 90° and 0° with mixing elements in forward stagger angles of 30° and 60° and one distributive feed screw element at the end of the granulator barrel in order to reduce the number of oversized agglomerates.

In the granulation process, the temperature of the jacket around the granulator barrel was kept constant at 25 °C using an active cooling system, and other parameters were set according to the designed experiments.

### 2.3. Experimental Design

I-optimal design was structured based on an iterative search algorithm and provided lower average prediction variance across the region of experimentation, which is desirable for response surface methods (RSMs) as prediction is important. In the current study, five process variables, screw speed (*X*_1_), L/S ratio (*X*_2_), powder feed rate (*X*_3_), number of the 60° mixing elements (*X*_4_), and number of the 30° mixing elements (*X*_5_), were systematically investigated to understand their effects on granule properties and optimize the granulation process. The common scale was utilized to describe each variable, whereby the highest coded value was equal to +1, the middle coded value was equal to 0, and the lowest was assigned a value of −1. Table 1 shows the independent variables and their levels in the DoE. The CQAs of TSWG were the moisture content, *D*_50_, span, and yield, and they were identified as *Y*_1_–*Y*_4_, respectively.

Considering that the independent variables included both continuous numeric variables (*X*_1_, *X*_2_, *X*_3_) and discrete numeric variables (*X*_4_, *X*_5_), an I-optimal design which contained 27 runs was performed, as shown in Table 2. The I-optimal design determined important factors and fit a quadratic polynomial model to the response. The results from the I-optimal design were adopted to build the nonlinear quadratic mathematical model, as follows:

(1) $$y=b_{0}+b_{1}X_{1}+b_{2}X_{2}+\cdots +b_{n}X_{n}+\sum b_{\mathrm{ik}}X_{i}X_{k}+\sum b_{\mathrm{ii}}X_{i}^{2}$$

In the equation, the measured response is denoted as *y*; *b*_0_ is a constant. The effect of each calculated term is described by the regression coefficients *b*_1_ to *b_i_*. Independent variable *X_i_* is coded in terms of factors. The interaction and quadratic terms are denoted as *X_i_X_k_* and *X_i_*^2^, respectively.

### 2.4. Characterization of Particle Properties

The granules exiting the system were collected and analyzed using different techniques to quantify the attributes. After drying for 24 h in a 60 °C oven, the samples were analyzed for particle size distribution, span of the size distribution (span), and granule yield. All measurements were performed in triplicate, and the test methods followed a previously published paper [18].

### 2.5. Method Validation

Validation studies were conducted to evaluate the process models using the percentage of prediction error, which was calculated with Equation (2).

(2) $$e=\frac{\left|y-\hat{y}\right|}{y}\%$$

### 2.6. Data Analysis and Modeling

Design-Expert 12 software (Stat-Ease Inc., Minneapolis, MN, USA) was utilized for conducting the five-factor, three-level I-optimal design in this study. Additionally, this software was employed for generating response contour plots and performing the relevant analyses.

## 3. Results and Discussion

### 3.1. Fitting Data to the Model

Table 3 displays an overview of the primitive data. The models were constructed using a forward/backward stepwise regression method and then regression of all subsets with the minimized Bayesian information criterion (BIC) to determine the model with the best prediction capability. The non-statistically significant effects were eliminated, and the quadratic polynomial equations were simplified during the regression analysis (Table 4). The regression models produced satisfactory results, with coefficients (R^2^) greater than 0.9. The predicted R^2^ values were reasonably close to the adjusted R^2^ values, indicating that the experimental data were well matched by the proposed models. All lack-of-fit tests were not significant relative to the pure error (*p*-value of the lack-of-fit test greater than 0.05), which means the results of the non-significant lack-of-fit test are good. The equations in terms of coded factors are listed in Equations (3)–(6), corresponding to *Y*_1_ to *Y*_4_, which can be used to make predictions about the response for given levels of each factor. By default, the high levels of the factors are coded as +1 and the low levels are coded as −1. The coded equation is useful for identifying the relative impact of the factors by comparing the factor coefficients.

### 3.2. The Effect of Factors on the Moisture Content

**Figure 2:**
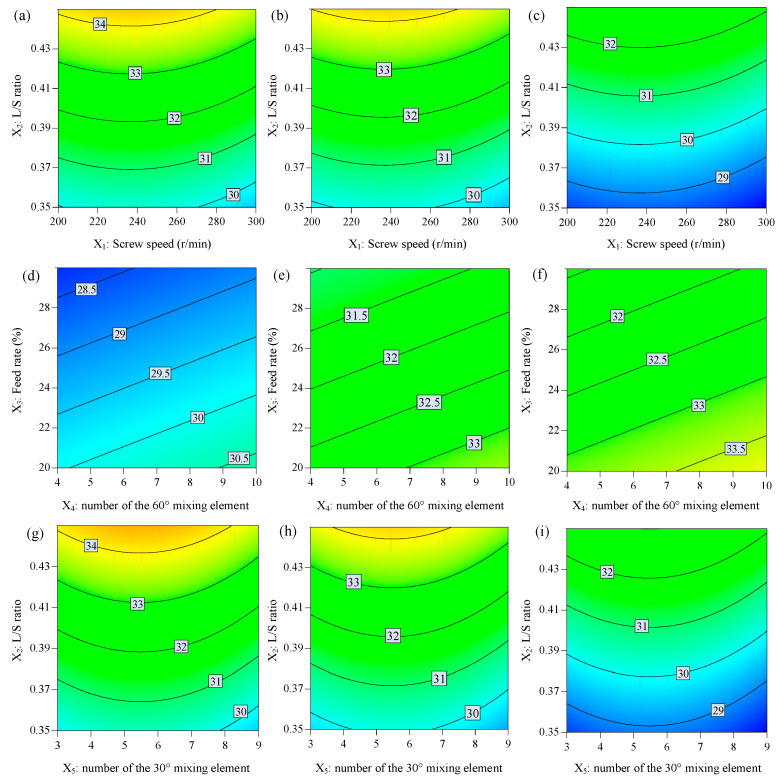
The response contour plot displays the effects of independent variables on the moisture content: (**a**–**c**) effects of *X*_1_ and *X*_2_, (**d**–**f**) *X*_3_ and *X*_4_, and (**g**–**i**) *X*_2_ and *X*_5_ on the moisture content while other factors were maintained at low, middle, and high levels, respectively.

Response contour plots were utilized for illustrating the correlation between the independent and dependent variables. These plots allow for the simultaneous examination of the impact of two factors on the response while maintaining the other factors constantly in a two-dimensional setting. By varying the axis variables while maintaining other factors at stationary levels, we observed the change rules intuitively, as shown in Figure 2. The regression equation and ANOVA results are shown in Table 4 and Table 5.

In this study, it can be observed from Table 5 that the factor screw speed (*X*_1_) and the quadratic term *X*_1_^2^ had negative effects on the moisture content (*Y*_1_). Similarly, the number of 60° kneading elements (*X*_5_) and the quadratic term *X*_5_^2^ had negative effects on the moisture content (*Y*_1_). The L/S ratio (*X*_2_) and number of 30° kneading elements (*X*_4_) had positive effects on the moisture content (*Y*_1_). The powder feed rate (*X*_3_) had negative effects on the moisture content (*Y*_1_).

The L/S ratio (*X*_2_) was found to be the most influential factor for moisture content (*Y*_1_). This makes sense since a higher L/S ratio (*X*_2_) provides a greater liquid amount, leading to greater liquid distribution and providing more surface wetting of granules [19]. However, under the assumption of uniform material mixing at a fixed L/S ratio (*X*_2_), increasing the screw speed and powder feed rate will not change the moisture content of particles theoretically. A possible reason is that the increased screw speed and powder feed rate result in insufficient mechanical dispersion between liquid and particles to form a homogenous liquid distribution [11].

### 3.3. The Effect of Factors on the Mean Particle Size

**Figure 3:**
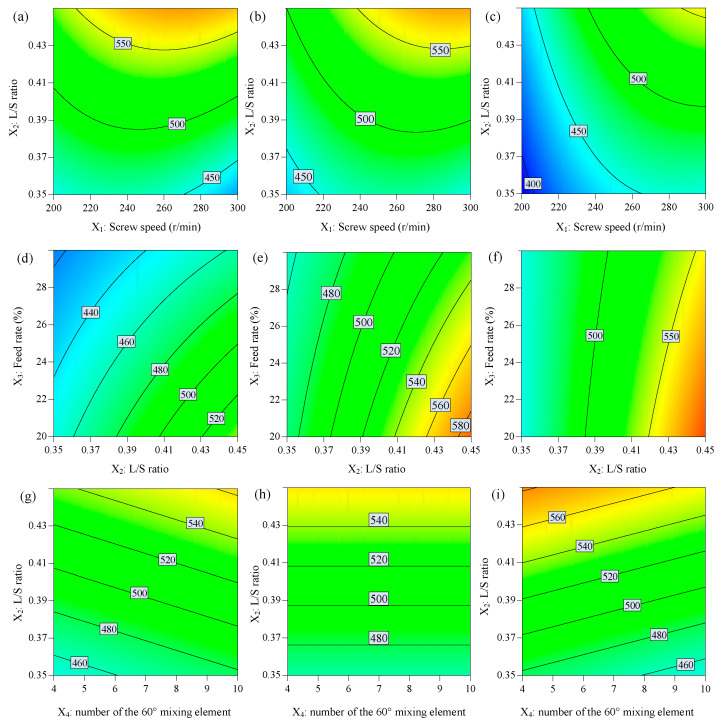
The response contour plot displays the effects of independent variables on the granule size D_50_: (**a**–**c**) effects of *X*_1_ and *X*_2_, (**d**–**f**) *X*_2_ and *X*_3_, and (**g**–**i**) *X*_2_ and *X*_4_ on the granule size D_50_ while other factors were maintained at low, middle, and high levels, respectively.

Particle size and distribution were regarded as some of the most important attributes of the granules because granules with appropriate particle size and distribution can significantly increase the blend uniformity, flowability, and compactibility of the product [8]. Response contour plots for D_50_ (*Y*_2_) are shown in Figure 3. Analysis of variance (ANOVA) was performed and results are shown in Table 4 and Table 5, which provide information about the model of D_50_ (*Y*_2_).

In this study, it can be observed from Table 4 that the factor screw speed (*X*_1_), L/S ratio (*X*_2_), and powder feed rate (*X*_3_) had significant effects on the mean granule size D_50_ (*Y*_2_). The L/S ratio (*X*_2_) was found to be the most influential factor in achieving granules with the desired mean granule size D_50_ (*Y*_2_), and the screw speed (*X*_1_) and L/S ratio (*X*_2_) had a positive effect on granule size. The powder feed rate (*X*_3_) had a negative effect on granule size. However, the number of 30° (*X*_4_) and the number of 60° kneading elements (*X*_5_) were not found as having a significant effect on granule size in this study. Interaction effects between *X*_1_ and *X*_2_, and *X*_1_ and *X*_3_ influenced the granule size positively, while interaction effects between *X*_2_ and *X*_3_, and *X*_3_ and *X*_4_ influenced the granule size negatively. The quadratic term *X*_1_^2^ had negative effects on D_50_ (*Y*_2_).

In this study, it can be seen that the higher screw speed can produce a larger granule diameter. It is reported that high screw speeds can increase the conveying capacity of the screws, resulting in a lower barrel fill level and lower residence times within the granulator [6,10,20]. However, this appears to contradict some studies, suggesting that the granule size slightly increased at low screw speeds [7]. They found that the lower barrel fill level results in low compaction and particle interaction and is not conducive to the growth of granules. However, at low screw speeds, the longer residence times of particles in the barrel allow for greater growth of the granules. A possible reason is that increasing the screw speed can increase the throughput and provide a greater compaction force, which enhances the consolidation and compaction between granules in the barrel; this could promote granulation and produce granules with larger sizes. However, at the same time, the intense mechanical forces generated by the high-speed rotation of the screws cause more aggressive particle breakage and attrition, resulting in smaller granules. These two effects are opposite for particle growth, and under different conditions, one of the factors may play a dominant role. It may be that, as reported within typical operation limits (e.g., screw speed and throughput ranges, kneading element configuration), screw speed has a positive effect on granule growth, but outside the upper and lower ranges, the effect on granule size becomes the opposite [10]. A possible reason is the high fill levels at low screw speeds, which lead to material compaction where blockages can form at high mass loads.

In the present study, the effect of the L/S ratio (*X*_2_) on particle size was in agreement with the research report that increasing the L/S ratio (*X*_2_) can reduce the number of fines and produce particles with superior flow properties [5,7]. A possible reason is that an increased L/S ratio (*X*_2_) can provide more liquid to form a higher liquid distribution and more surface wetting of granules [19].

It is found that increasing material feed rate (*X*_3_) at a constant L/S ratio (*X*_2_) mainly had a negative effect on the particle size of granules. That is, an increasing throughput decreased the average granule diameter in the present study. It is explained that the higher barrel fill level at high throughput leads to restricted liquid distribution along with an increase in friction between the barrel wall and granules, resulting in higher attrition of granules [21]. However, some research showed that the average granule diameter was higher at increased material throughput due to a higher filling degree of the barrel [7,9], and this may be attributed to the presence of a kneading screw element in the screw configuration, which could promote granulation.

It was observed that the number of 30° and 60° kneading elements, as well as their interaction, were found to have no significant effect on the granule size. A possible reason was assumed that the increased number of kneading elements provided stronger mixing ability and compaction forces, which enhanced the consolidation and compaction between granules and also led to a more homogeneous distribution of the granulation liquid within the setting range [11].

### 3.4. The Effect of Factors on the Span

**Figure 4:**
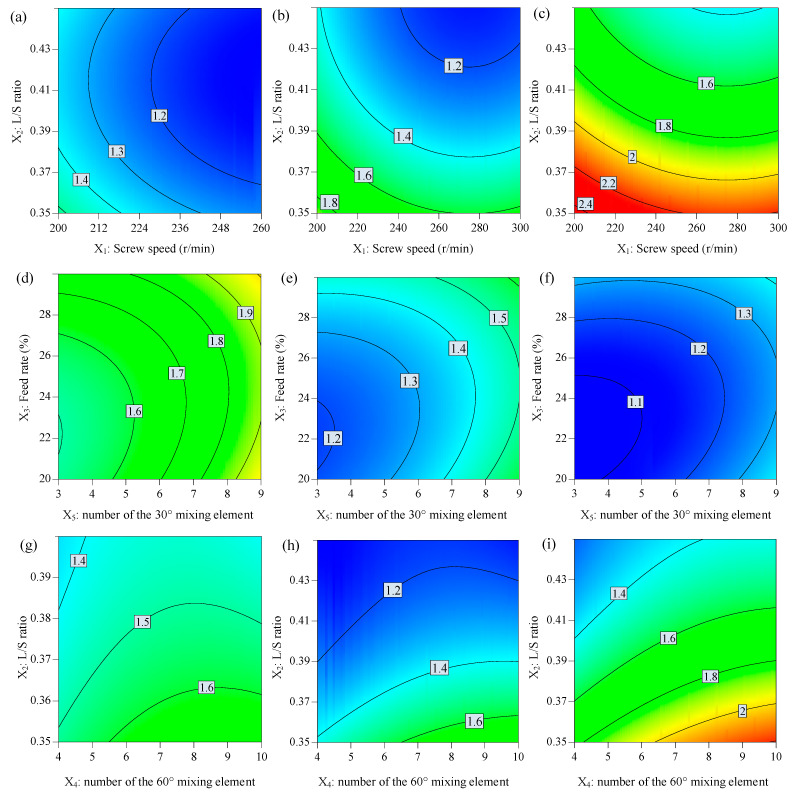
The response contour plot displays the effects of independent variables on the span: (**a**–**c**) effects of *X*_1_ and *X*_2_, (**d**–**f**) *X*_3_ and *X*_5_, and (**g**–**i**) *X*_2_ and *X*_4_ on the span while other factors were maintained at low, middle, and high levels, respectively.

The results of the ANOVA analysis of span (*Y*_3_) are shown in Table 4 and Table 5. Response contour plots are shown in Figure 4. The results showed that screw speed (*X*_1_), L/S ratio (*X*_2_), and powder feed rate (*X*_3_) had significant impacts on span (*Y*_3_), and the L/S ratio (*X*_2_) was found to be the most significant term among them. The three main terms were reported to be critical factors that determine the granule properties [3]. These parameters have a great influence on the fill level within the granulator. The variation in fill level can make considerable differences in binder distribution and granule properties [5,22]. The effect of the L/S ratio (*X*_2_) on the span is similar to its effect on D_50_ (*Y*_2_). An appropriate amount of liquid is a necessary condition for particles to grow to the target size and distribution. Within the scope of the experiment, a higher L/S ratio (*X*_2_) can produce granules with a more uniform distribution. The number of 30° (*X*_4_) and 60° kneading elements (*X*_5_), as well as their interaction, were found to have positive significant effects on the span (*Y*_3_), which indicates that increasing *X*_5_ can broaden the span of the final granules. Kneading elements can make the liquid distribution become more uniform in screw configurations and provide more densification [23]. We found a notably higher improvement in liquid distribution homogeneity when increasing the kneading block length [24]. When the number of kneading elements increased, it can provide stronger compaction forces, which enhanced the consolidation and compaction between the particles. The uniform distribution during nucleation makes the granules’ growth less reliant on mechanical dispersion provided by the kneading elements [24].

### 3.5. The Effect of Factors on the Production Yield

**Figure 5:**
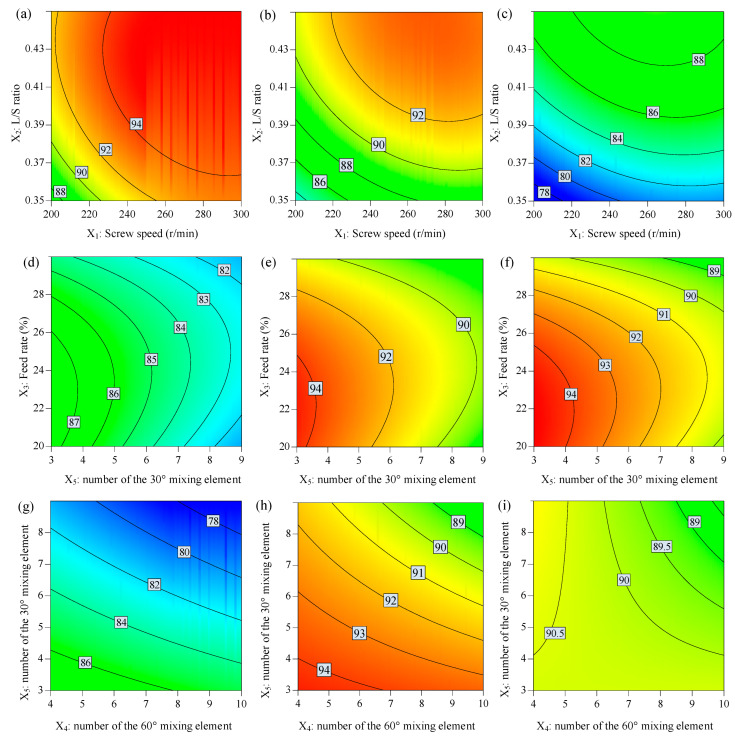
The response contour plot displays the effects of independent variables on the production yield: (**a**–**c**) effects of *X*_1_ and *X*_2_, (**d**–**f**) *X*_3_ and *X*_5_, and (**g**–**i**) *X*_4_ and *X*_5_ on the production yield while other factors were maintained at low, middle, and high levels, respectively.

Table 4 and Table 5 display the results from the ANOVA analysis on yield (*Y*_4_). Response contour plots are shown in Figure 5. It can be observed from Table 5 that the screw speed (*X*_1_) and L/S ratio (*X*_2_) had positive effects on the production yield. The powder feed rate (*X*_3_), the number of 30° (*X*_4_), and the number of 60° kneading elements (*X*_5_) had negative effects on the production yield. There was an interaction between factors *X*_1_*X*_2_, *X*_1_*X*_3_, and *X*_4_*X*_5_, which had a negative effect on yield (*Y*_4_), while *X*_2_*X*_4_, *X*_2_*X*_5_, and *X*_3_*X*_5_ had a positive effect on yield (*Y*_4_). Quadratic terms *X*_1_^2^, *X*_2_^2^, and *X*_3_^2^ had a negative effect on yield (*Y*_4_).

The L/S ratio (*X*_2_) was the most significant positive factor that contributed to the production yield model. It was seen that the yield increased as the L/S ratio (*X*_2_) increased, and granules produced at a low L/S ratio (*X*_2_) had a high span and broad size distribution, including the fine and large agglomerates. However, granules produced at a high L/S ratio (*X*_2_) had low span and narrow size distribution [20]. It is inferred that the low L/S ratio (*X*_2_) results in concentrated wetted areas by direct injection through liquid inlet ports. The particles suffer from insufficient mechanical dispersion to form homogenous liquid distribution, which results in small dry fine and large wetted agglomerates [25].

### 3.6. Defining a Design Space and Validation of the TWSG Process

In order to produce granule products that meet the quality requirements, the TWSG process parameters should be operated within a defined design space. The design space represents a combination and interaction of input variables and process parameters that are designed to meet the quality attributes [26]. Therefore, the process parameters and the screw element configuration can be optimized to produce a granular product with different physical properties.

**Figure 6:**
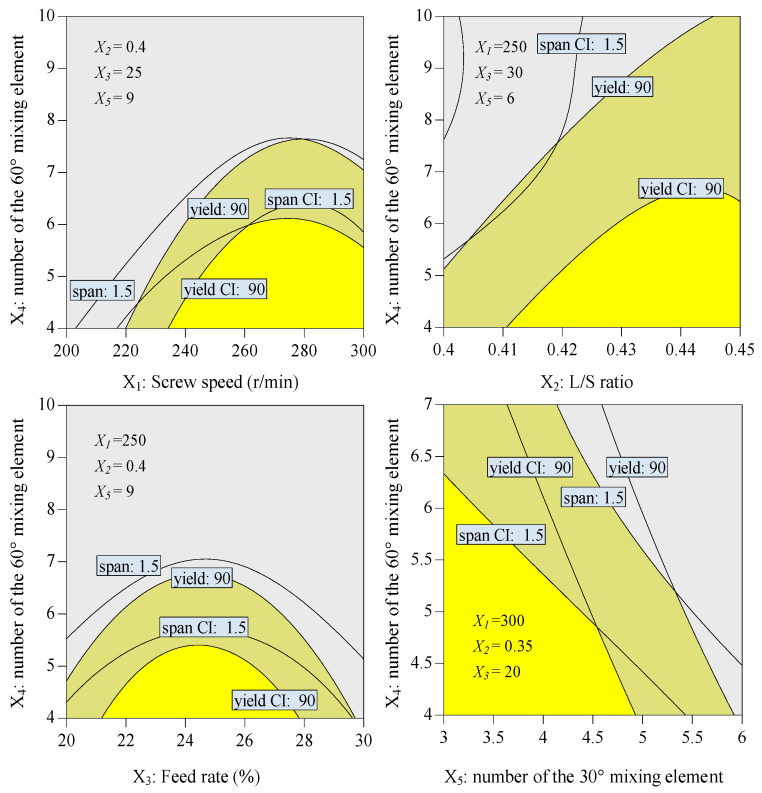
Design space for TSWG from different viewpoints.

The granules obtained by TSWG need to be dried downstream; in the drying process, the amount of water needed to be reduced during the drying phase. To maximize production efficiency and minimize energy consumption, it was encouraging to produce qualified products with minimum water [8]. Therefore, the minimal L/S ratio required for granulation needs to be determined. The granules with a *span* value less than 1.50 were considered to have a narrow size distribution that can enable efficient downstream tableting processing. Meanwhile, the median particle size D_50_ needs to be close to the desired granule size, and the particle size distribution should be as narrow as possible to obtain the maximum yield. Therefore, the granule diameter (400 μm < D_50_ < 600 μm), span (span < 1.5), and production yield (150 μm < granule size < 1200 μm, target: maximize) were used as representative CQAs of granules in the design space determination based on the previous experiments and data reported in the literature [8,27]. Table 6 displays the constraints regarding the CQAs. We navigated the design space via the regression models of D_50_, span, and production yield. Figure 6 illustrates the design space from various perspectives.

The overlay plots depict the range of two factors while the remaining factors are held at a specified level. The bright yellow section represents the design space for TSWG with a 90% confidence interval. The dark yellow section indicates a portion of the design space where there is a 10% probability of failing to meet process objectives. The dark gray area is outside of the design space and does not meet technical requirements. The successful application of the I-optimal design helped define the design space, ensuring the desired quality of the final granule by considering the operating parameter ranges of the five factors being investigated.

Additional validation experiments were carried out to evaluate the accuracy of the established regression models. The operating parameters of one experiment were within the design space, and the other experimental parameters were outside the design space. The operating conditions and experimental and predicted values are shown in Table 7. The results showed that the prediction error percentage, comparing the actual values obtained from validation experiments with the anticipated values from the responses, was below 10%, which is considered acceptable. This suggests that the models are reliable and possess an effective predictive capability.

## 4. Conclusions

The results of this study demonstrate the effective application of the I-optimal design method in analyzing the factors influencing TWSG related to target granule attributes. Five investigated process parameters—screw speed, L/S ratio, powder feed rate, and numbers of the 60° and 30° mixing elements—were studied via modeling using the I-optimal design. A design space that defined the ranges of operating parameters for TWSG was determined based on constraints for the target granule quality: mean particle size, span, and yield. Validation experiments confirmed the accuracy and reliability of the mathematical models. The application of the I-optimal design provides a precise estimation of the parameters and prediction of the response to determine optimum operating conditions. This study enhances comprehension of TWSG cost-effectively and efficiently, confirming that an I-optimal design is a viable approach for exploring the complicated TWSG process.

## Figures and Tables

**Table 1:** Independent variables and their levels in I-optimal design.

Independent Variables		Levels		
		Minimum (−1)	Intermediate (0)	Maximum (+1)
Continuous	*X*_1_: screw speed (r/min)	200	250	300
	*X*_2_: L/S ratio	0.35	0.4	0.45
	*X*_3_: powder feed rate (%)	20	25	30
Discrete numeric	*X*_4_: number of the 60° mixing elements (pcs)	4	7	10
	*X*_5_: number of the 30° mixing elements (pcs)	3	6	9

**Table 2:** Independent variable sets in I-optimal design and responses.

Run Order	Pattern	Independent Variables				
		*X*_1_	*X*_2_	*X*_3_	*X*_4_	*X*_5_
1	0--++	250	0.35	20	10	9
2	0----	250	0.35	20	4	3
3	+-0-+	300	0.35	25	4	9
4	0+-+0	250	0.45	20	10	6
5	-++00	200	0.45	30	7	6
6	0-00-	250	0.35	25	7	3
7	+++-0	300	0.45	30	4	6
8	--0+-	200	0.35	25	10	3
9	-0-0-	200	0.4	20	7	3
10	-00++	200	0.4	25	10	9
11	+0--+	300	0.4	20	4	9
12	+-+0-	300	0.35	30	7	3
13	00+-+	250	0.4	30	4	9
14	--+0+	200	0.35	30	7	9
15	-+--+	200	0.45	20	4	9
16	00000	250	0.4	25	7	6
17	+-++0	300	0.35	30	10	6
18	00000	250	0.4	25	7	6
19	+0-+-	300	0.4	20	10	3
20	00000	250	0.4	25	7	6
21	++0++	300	0.45	25	10	9
22	0+++-	250	0.45	30	10	3
23	-0+--	200	0.4	30	4	3
24	0--00	250	0.35	20	7	6
25	--0-0	200	0.35	25	4	6
26	+---0	300	0.35	20	4	6
27	0+0--	250	0.45	25	4	3

**Table 3:** The responses of I-optimal design.

NO.	*Y*_1_: Moisture Content	*Y*_2_: *D*_50_	*Y*_3_: Span	*Y*_4_: Yield
	%	μm		%
1	30.49	498.5	2.16	81.19
2	31.74	463.1	1.24	92.12
3	28.14	450.9	1.58	88.18
4	35.86	609.1	1.14	92.20
5	33.57	441.9	1.60	88.64
6	29.50	498.6	1.37	91.31
7	32.53	592.0	1.24	91.58
8	29.65	438.3	1.74	84.82
9	32.20	481.8	1.48	90.12
10	30.78	464.4	1.88	85.00
11	31.22	476.8	1.42	90.87
12	27.90	446.9	1.71	86.61
13	29.63	485.6	1.47	89.45
14	28.39	389.6	2.25	78.37
15	33.47	555.4	1.38	89.27
16	32.19	508.4	1.30	92.73
17	29.12	454.9	1.87	83.81
18	31.99	521.9	1.22	90.81
19	32.02	515.4	1.11	95.05
20	32.28	528.5	1.34	91.86
21	33.57	586.5	1.32	90.08
22	32.80	517.2	1.36	91.13
23	30.67	461.8	1.54	88.40
24	30.52	438.9	1.73	86.66
25	29.50	438.0	1.68	85.38
26	29.01	440.0	1.53	89.37
27	32.80	536.2	1.24	93.65

**Table 4:** The regression equation of the response surface quadratic model and their ANOVA results.

Response Variable	Regression Equation		R ^2^	*Adj* R^2^ *	*Pre* R^2^ *	*p* (Lack-of-Fit Test)	PRESS *
*Y*_1_	=32.15 − 0.25*X*_1_ + 2.07*X*_2_ − 0.86*X*_3_ + 0.33*X*_4_ − 0.23*X*_5_ − 0.46*X*_1_^2^ − 0.67*X*_5_^2^	(3)	0.9402	0.9182	0.8740	0.0615	12.47
*Y*_2_	=512.16 + 22.95*X*_1_ + 47.68*X*_2_ − 18.24*X*_3_ + 14.75*X*_1_*X*_2_ + 21.91*X*_1_*X*_3_ − 9.96*X*_2_*X*_3_ − 13.30*X*_3_*X*_4_ − 22.42*X*_1_^2^	(4)	0.9072	0.8659	0.7844	0.2183	16088.62
*Y*_3_	=1.31 − 0.12*X*_1_ − 0.23*X*_2_ + 0.068*X*_3_ + 0.088*X*_4_ + 0.14*X*_5_ − 0.062*X*_2_*X*_4_ − 0.11*X*_2_*X*_5_ − 0.054*X*_3_*X*_5_ + 0.069*X*_4_*X*_5_ + 0.12*X*_1_^2^ + 0.093*X*_2_^2^ + 0.11*X*_3_^2^ − 0.058*X*_4_^2^ + 0.051*X*_5_^2^	(5)	0.9706	0.9364	0.7750	0.4604	0.4937
*Y*_4_	=91.90 + 1.85*X*_1_ + 2.99*X*_2_ − 1.09*X*_3_ − 1.17*X*_4_ − 2.06*X*_5_ − 0.62*X*_1_*X*_2_ − 0.38*X*_1_*X*_3_+ 0.51*X*_2_*X*_4_ + 0.92*X*_2_*X*_5_ + 0.77*X*_3_*X*_5_ − 0.66*X*_4_*X*_5_ − 1.54*X*_1_^2^ − 1.54*X*_2_^2^ − 1.55*X*_3_^2^	(6)	0.9791	0.9548	0.8633	0.7291	52.71

* *Adj* R^2^: Adjusted R^2^; *Pre* R^2^: Predicted R^2^; and PRESS: Predicted residual sum of squares.

**Table 5:** Regression coefficients and associated probability values (*p*-values) for the models of granule CQAs.

Model Terms	*Y*_1_*:* Moisture Content		*Y*_2_*:* D_50_		*Y*_3_*:* Span		*Y*_4_*:* Yield	
	Coef.	*p*-Values	Coef.	*p*-Values	Coef.	*p*-Values	Coef.	*p*-Values
Intercept	32.15		512.16		1.31		91.90	
*X*_1_	−0.25	0.0968	22.95	0.0003	−0.12	<0.0001	1.85	< 0.0001
*X*_2_	2.07	<0.0001	47.68	<0.0001	−0.23	<0.0001	2.99	<0.0001
*X*_3_	−0.86	<0.0001	−18.24	0.0017	0.07	0.0043	−1.09	0.0003
*X*_4_	0.33	0.0215			0.09	0.0003	−1.17	<0.0001
*X*_5_	−0.23	0.1070			0.14	<0.0001	−2.06	<0.0001
*X*_1_ *X*_2_			14.75	0.0364			−0.62	0.0477
*X*_1_ *X*_3_			21.91	0.0026			−0.38	0.2213
*X*_1_ *X*_4_								
*X*_1_ *X*_5_								
*X*_2_ *X*_3_			−9.96	0.1270				
*X*_2_ *X*_4_					−0.06	0.0131	0.51	0.069
*X*_2_ *X*_5_					−0.11	0.0008	0.92	0.0066
*X*_3_ *X*_4_			−13.30	0.0463				
*X*_3_ *X*_5_					−0.05	0.0413	0.77	0.015
*X*_4_ *X*_5_					0.07	0.0070	−0.66	0.0171
*X*_1_^2^	−0.46	0.0569	−22.42	0.0133	0.12	0.0033	−1.54	0.001
*X*_2_^2^					0.09	0.0140	−1.54	0.0012
*X*_3_^2^					0.11	0.0044	−1.55	0.001
*X*_4_^2^					−0.06	0.1333		
*X*_5_^2^	−0.67	0.0085			0.05	0.1302		

**Table 6:** Constraints on dependent responses.

Dependent Responses	Constraints	Optimum
*X*_1_: L/S ratio	0.35 ≤ *X*_1_ ≤ 0.45	minimum
*Y*_2_: Mean particle size *D*_50_	400 μm ≤ *Y*_2_ ≤ 600 μm	-
*Y*_3_: Span of granule	*Y*_3_ ≤ 1.5	minimum
*Y*_4_: Yield	90 ≤ *Y*_4_ ≤ 100	maximize

**Table 7:** Process parameters and experimental results of validation test points.

CPP (*X*_1_; *X*_2_; *X*_3_; *X*_4_; *X*_5_)	CQA	Experimental Value (y)	$\mathrm{Predicted}\;\mathrm{Value}\;(\hat{y})$	ARD (%)
(240; 0.45; 30; 4; 7)	*Y*_1_	33.1	30.5	7.7
	*Y*_2_	532.1	494.2	7.1
	*Y*_3_	1.3	1.3	5.2
	*Y*_4_	91.2	97.4	6.8
(200; 0.4; 25; 7; 7)	*Y*_1_	33.2	31.8	4.3
	*Y*_2_	466.8	499.1	6.9
	*Y*_3_	1.6	1.5	4.9
	*Y*_4_	87.8	94.8	7.9

## Data Availability

All data have been reflected in the paper, and no additional data are required.
